# Advancing 100m sprint performance prediction: A machine learning approach to velocity curve modeling and performance correlation

**DOI:** 10.1371/journal.pone.0303366

**Published:** 2024-05-13

**Authors:** Chung Kit Tam, Zai-Fu Yao

**Affiliations:** 1 Department of Kinesiology, National Tsing Hua University, Hsinchu City, Taiwan; 2 College of Education, National Tsing Hua University, Hsinchu City, Taiwan; 3 Research Center for Education and Mind Sciences, National Tsing Hua University, Hsinchu City, Taiwan; 4 Basic Psychology Group, Department of Educational Psychology and Counseling, National Tsing Hua University, Hsinchu City, Taiwan; University of Montenegro, MONTENEGRO

## Abstract

This study presents a novel approach to modeling the velocity-time curve in 100m sprinting by integrating machine learning algorithms. It critically addresses the limitations of traditional speed models, which often require extensive and intricate data collection, by proposing a more accessible and accurate method using fewer variables. The research utilized data from various international track events from 1987 to 2019. Two machine learning models, Random Forest (RF) and Neural Network (NN), were employed to predict the velocity-time curve, focusing on the acceleration phase of the sprint. The models were evaluated against the traditional exponential speed model using Mean Squared Error (MSE), with the NN model demonstrating superior performance. Additionally, the study explored the correlation between maximum velocity, the time of maximum velocity occurrence, the duration of the maximum speed phase, and the overall 100m sprint time. The findings indicate a strong negative correlation between maximum velocity and final time, offering new insights into the dynamics of sprinting performance. This research contributes significantly to the field of sports science, particularly in optimizing training and performance analysis in sprinting.

## Introduction

The 100m sprint, a cornerstone event in track and field, has been extensively analyzed to understand and model the dynamics of velocity [1]. Recent advancements have led to more sophisticated approaches, including the use of radar data to examine the interplay between fatigue and lower limb properties [2] and radio-based tracking systems to validate sprint parameters [3]. While traditional models have segmented the sprint into distinct phases [3–6], these models often require precise, sometimes difficult to measure data, and may not accurately represent the early acceleration and deceleration phases [3, 7, 8]. In response to these limitations, this study explores the application of machine learning (ML) techniques, which have seen increasing use in sports analytics [9, 10] to offer a more accessible and accurate modeling of sprinting velocity curves.

Studying the 100m sprint is crucial for understanding human behavior in sports because it combines physical abilities with psychological and strategic elements. The sprint requires a complex blend of speed, strength, technique, and mental focus. Researchers gain insights into how these elements interact by analyzing the velocity-time curve in the 100m sprint using machine learning algorithms [2, 7]. Furthermore, the sprint is divided into distinct phases, each with unique characteristics that contribute to overall performance [4–6]. As demonstrated in the study, predicting sprint performance using machine learning approaches is significant as it offers a more accessible and accurate method of analyzing sprint dynamics. This is especially relevant for training and injury prevention, where understanding the nuances of each phase can lead to more effective training regimens and lower injury risks [11, 12]. Additionally, the correlation between different sprint parameters and final sprint times provides valuable insights into the factors that most significantly impact sprint performance, thereby guiding athletes and coaches in their training focus.

Since the past decade, the equation of velocity to time curve has been proposed [1, 13] more and more research has extended from the original equation. For example, Morin et al. (2006) based on the data from radar and modeled the velocity-time curve to investigate the relationship between fatigue and runners’ lower limb properties [2]. Seidl et al. (2021) used data from a radio-based tracking system and a basic speed model to validate sprint parameters in the top speed interval [3]. Moreover, many researchers have investigated how 100m sprinting can be split into different phases and what characteristics have been shown in different phases [4, 14]. These studies highlighted the evolution of understanding in sprinting dynamics, emphasizing the importance of accurate data collection and applying advanced modeling techniques, such as machine learning, to refine velocity-time curves. Hence, we aimed to use machine learning algorithms to simplify modifying the velocity curve in 100m sprinting with fewer constants and explore relationships between various sprinting parameters and overall performance.

**Fig 1:**
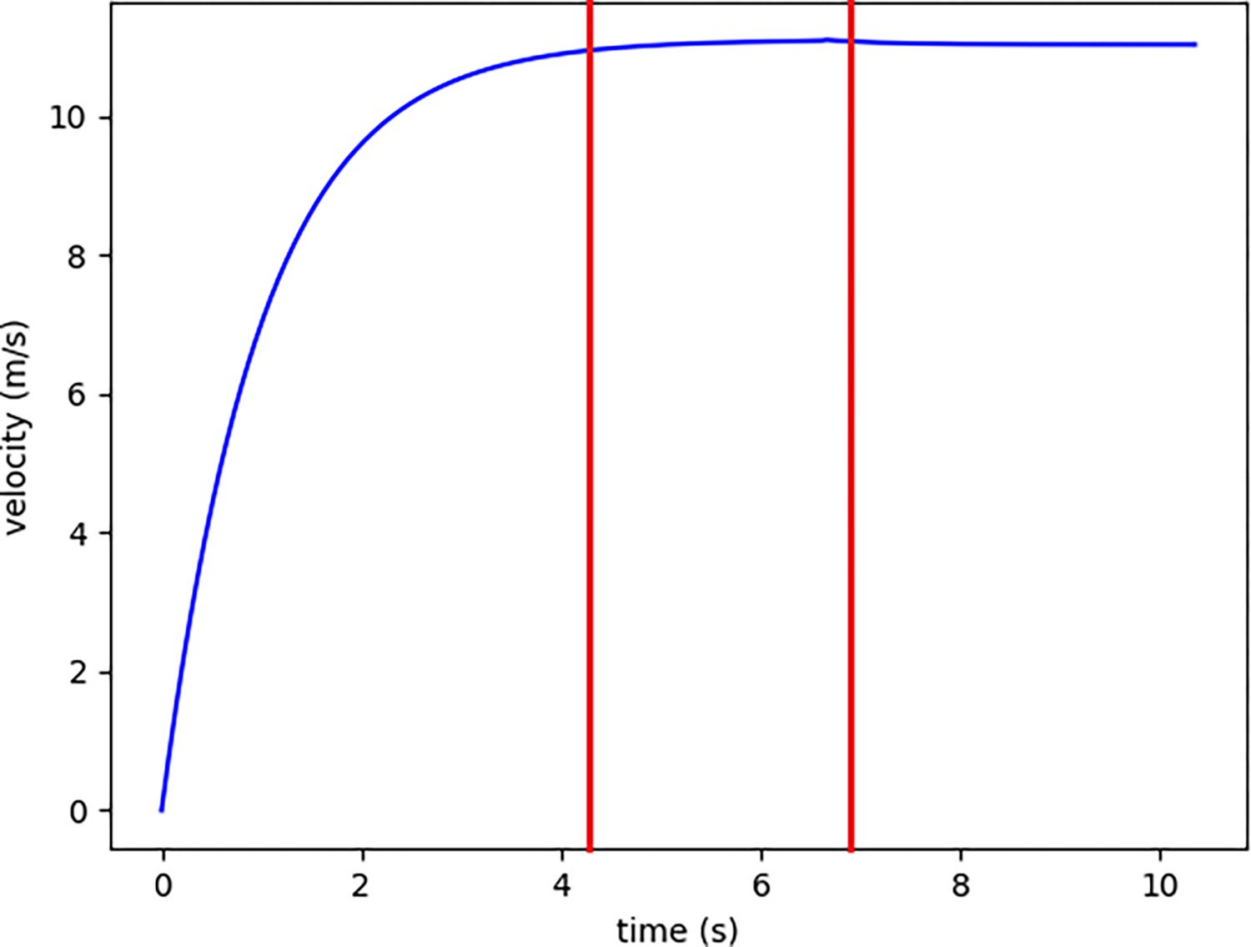
The modeled velocity to time curve of actual data, and the red line divides the v(t) curve into 3 parts.

Prior to this research, the prevalent approach in modeling velocity-time curves in athletics, particularly for the 100m sprint, heavily relied on biexponential functions, a method introduced by [2, 7] and subsequently adopted by others. These models, while innovative, presented notable challenges, including the requirement for highly accurate data collection and the complex measurement of specific constants. Conceptually, a velocity curve is expected to depict three distinct stages: the acceleration, peak speed, and deceleration phases, as described in prior studies [15–19] (Fig 1). However, these conventional models demonstrated several shortcomings. Primarily, they depended heavily on precise instrument-derived data. This observation is evident in previous works [3, 7] where adjustments to the speed model constants were necessary based on a comparison with actual data using the least squares method. Additionally, certain constants within these models proved challenging to quantify accurately, such as those in the Arsac and Locatelli (2002) model, which required the inclusion of factors like the energy cost of movement, air resistance, and the efficiency of metabolic to external work conversion [8]. A critical finding from our data analysis was the poor performance of these models in accurately capturing the initial 10 meters of a sprint, alongside unclear deceleration rates in certain instances. The primary issue with these earlier models was their limited applicability in everyday training scenarios. Prior to this manuscript, there seemed to be a gap in understanding how ML can enhance the modeling of sprinting velocity curves, particularly in contrast to bi-exponential models. The hypothesis, therefore, is that ML can provide a more efficient and accurate framework for modeling 100m sprint velocity curves, requiring fewer variables and offering better fits for key phases of sprinting. This hypothesis is grounded in the limitations of existing models and the potential of ML, as indicated by the cited references.

Machine Learning (ML) offers a sophisticated approach to deciphering the relationship between independent variables and prediction targets in various domains, including sports [9, 10]. Its applications in sports, such as motor tracking, injury prediction, and athlete performance forecasting, are well-documented [11, 12, 20]. However, these models face challenges, including the need for precise data collection and difficulty in measuring certain variables. Our research aims to evaluate the hypothesis that ML, with its advanced data processing capabilities, can more effectively model 100m sprint velocity curves than traditional biexponential speed models. We posit that ML models require fewer constants while offering a more accurate representation of initial acceleration and deceleration phases. This study focuses on using ML for simpler modifications of velocity curves and investigating the interplay between various sprinting parameters. We anticipate uncovering critical insights into the relationships among maximum speed velocity, the timing of its occurrence, the duration of the maximum speed phase, and the overall 100m sprint time. This hypothesis builds upon the foundational understanding of traditional biexponential speed models provided by Morin et al. (2006) [2] while integrating the contemporary insights into ML’s utility in sports performance analysis as discussed by Horvat and Job (2020) [10] and the applicability of linear random forest algorithms in ML for modeling complex, non-linear targets like sprinting velocity, as explored by Ao et al. (2019) [21].

## Methods

### Data curation

Data was collected from athletefirst.org, including data from men’s 100m heats, semi-finals, and finals of the Olympic Games, World Athletic Championships, Asian Games, and even some domestic competitions. Data was recorded only from 1987 to 2019 due to COVID-19. Data without a split time of every 10m was excluded. Numbers replaced the names of athletes. Each split time is divided by 10m to get the average velocity of every 10m (from V_0−10_ to V_90−100_).

### Exponential speed model

**Fig 2:**
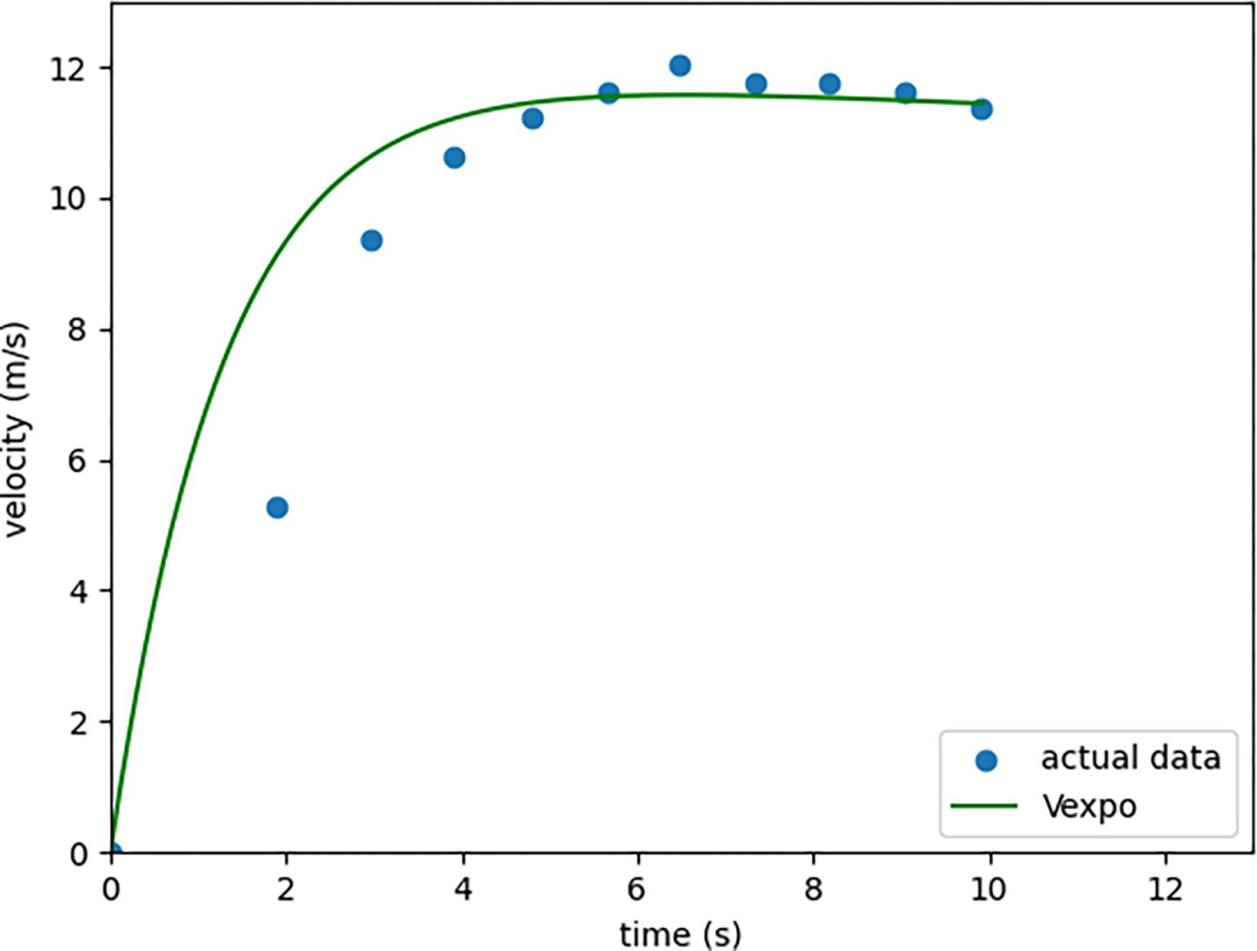
The sample of the traditional exponential speed model applied to our actual velocity data.

Our competitive tradition model is modified by Morin et al. (2006) [2], which is a biexponential function (Fig 2).


$$v(t)=V_{\max}[e^{\left(\frac{-t+tvmax}{\tau 2}\right)}-e^{\left(\frac{-t}{\tau 1}\right)}]$$ (1)

The velocity function of the time (Eq 1) curve included several variables. *V*_max_ is the maximum velocity collected by instruments. tvmax is the time to reach maximum velocity. *τ*2 and *τ*1 are the constants for the acceleration and deceleration in this equation, respectively.

### Machine learning model

To improve predictions for the acceleration phase, we compared two different ML algorithms for generating the *v*(*t*) curve: random forest (RF) and neural network (NN). In the study of Horvat and Job (2020) [10], the most popular ML algorithm was a neural network. Additionally, based on Ao et al. (2019) study [21], the linear random forest algorithm is more suitable when applied to nonlinear targets.

In our study, the input variables are maximum velocity (*V*_*max*_) and the final time of 100m (*T*_*F*_). We chose these two variables because they are easy to collect in training. As mentioned before, the biggest problem with the previous speed model is the inconvenience of collecting independent variables. The response variables are from V_0−10_ to V_90−100_, 10 variables in total. Validation for both machine learning models is done using leave-one-out cross-validation.

### Performance analysis

Mean squared error (MSE) will be used to compare the accuracy of the exponential model and the two ML models. The equation of MSE:

$$MSE=\frac{\Sigma {\left(y_{i}-p_{i}\right)}^{2}}{n}$$ (2)

In constructing our Machine Learning (ML) models and conducting analysis, we employed several Python modules, each serving a distinct purpose in our methodology. NumPy was utilized for efficient array operations, forming our numerical computations’ basis. For data manipulation and organization, we used Pandas, a library well-suited for handling structured data.

The ML models were built using RandomForestRegressor and MLPRegressor from the scikit-learn library, chosen for their robustness in handling regression tasks. To quantify the performance of our models, we computed the Mean Squared Error (MSE) and the coefficient of determination (*r*^2^) using scikit-learn’s metrics module. *r*^2^ is also our index of effect size. According to Sullivan and Feinn (2012) [22], *r*^2^ of 0.04, 0.25 and 0.64 are considered as the small effect size, medium effect size, and large effect size, respectively.

Visualization of the velocity-time (v(t)) graphs was accomplished with Matplotlib, a versatile plotting library. Additionally, scikit-learn’s model_selection module provided LeaveOneOut cross-validation techniques, ensuring a thorough assessment of our models’ performance.

For optimization and interpolation tasks, we used the minimize function from scipy.optimize and interp1d from scipy.interpolate, respectively. These functions allowed us to refine our models’ parameters and interpolate data points for more accurate predictions.

The joblib library was included for saving and loading our model states, ensuring reproducibility and efficiency in our workflow. Lastly, for decision tree visualization, we used plot_tree from scikit-learn, and Seaborn was our choice for advanced statistical graphics.

**Fig 3:**
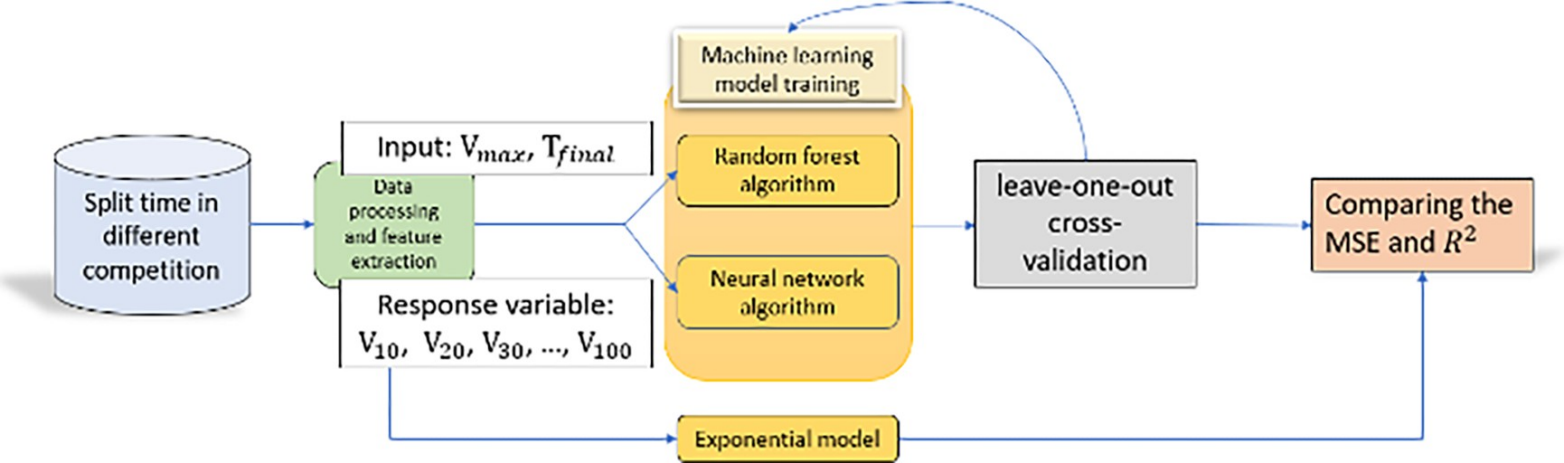
Flowchart from data collection to the prediction performance comparison.

Finally, all processes in the methodology section of the model are shown below (Fig 3).

### Correlation analysis

After the best algorithm is chosen, three parameters will be extracted from the modeled v(t) curves: maximum velocity, time of maximum velocity appearance, and length of maximum velocity phase. The final time will be kept using our original data. Pearson correlation coefficients are calculated using the Python module ‘pandas.DataFrame.corr’ in our study. The maximum velocity phase is defined as the maximum velocity multiplied by 0.98. The deceleration of 2% is based on the study by Mackala [5, 6].

## Results

**Fig 4:**
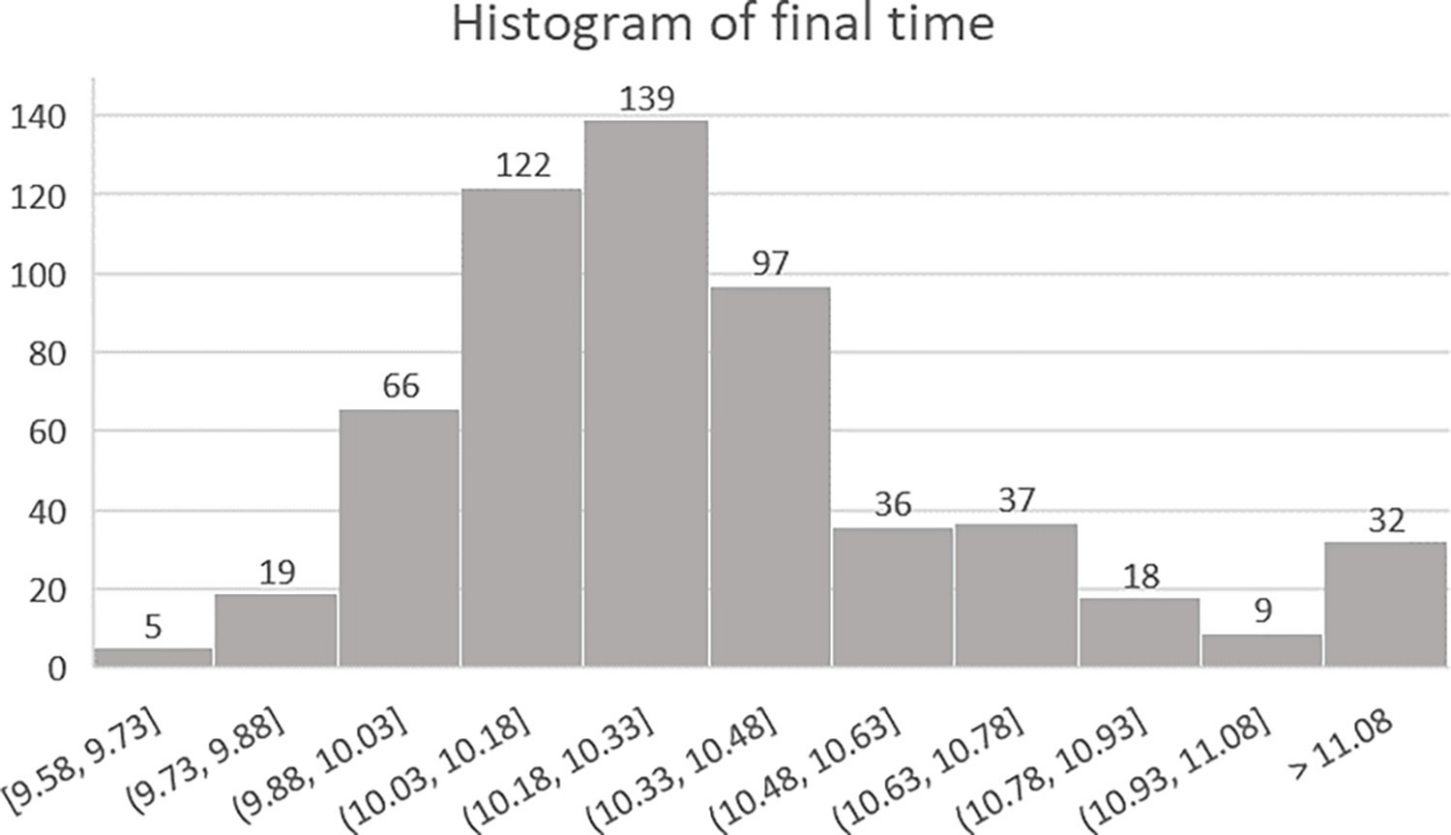
Histogram of final time in our data. It showed a right-skewed distribution.

After the data process, 580 data were utilized, and details of the data are shown in Table 1. The frequency distribution is shown in Fig 4.

**Table 1:** 

	T_*F*_	V_*max*_	V_0−10_	V_10−20_	V_20−30_	V_30−40_	V_40−50_	V_50−60_	V_60−70_	V_70−80_	V_80−90_	V_90−100_
n	580											
Mean	10.336	11.314	5.187	9.418	10.458	10.914	11.143	11.201	11.174	11.034	10.863	10.596
Standard deviation	0.355	0.509	0.174	0.284	0.373	0.383	0.425	0.46	0.477	0.515	0.548	0.608

**Fig 5:**
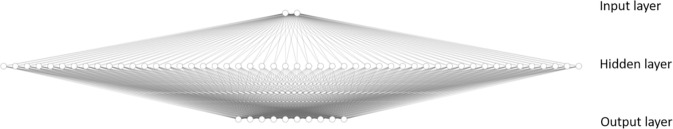
Description of the neural network model. Only one hidden layer with 50 neurons using "rectified linear units" (ReLU).

The trial-and-error method is used for both machine learning models to determine the parameters during model training. In the NN model, it consists of 2 inputs with 50 nodes (Fig 5).

**Fig 6:**
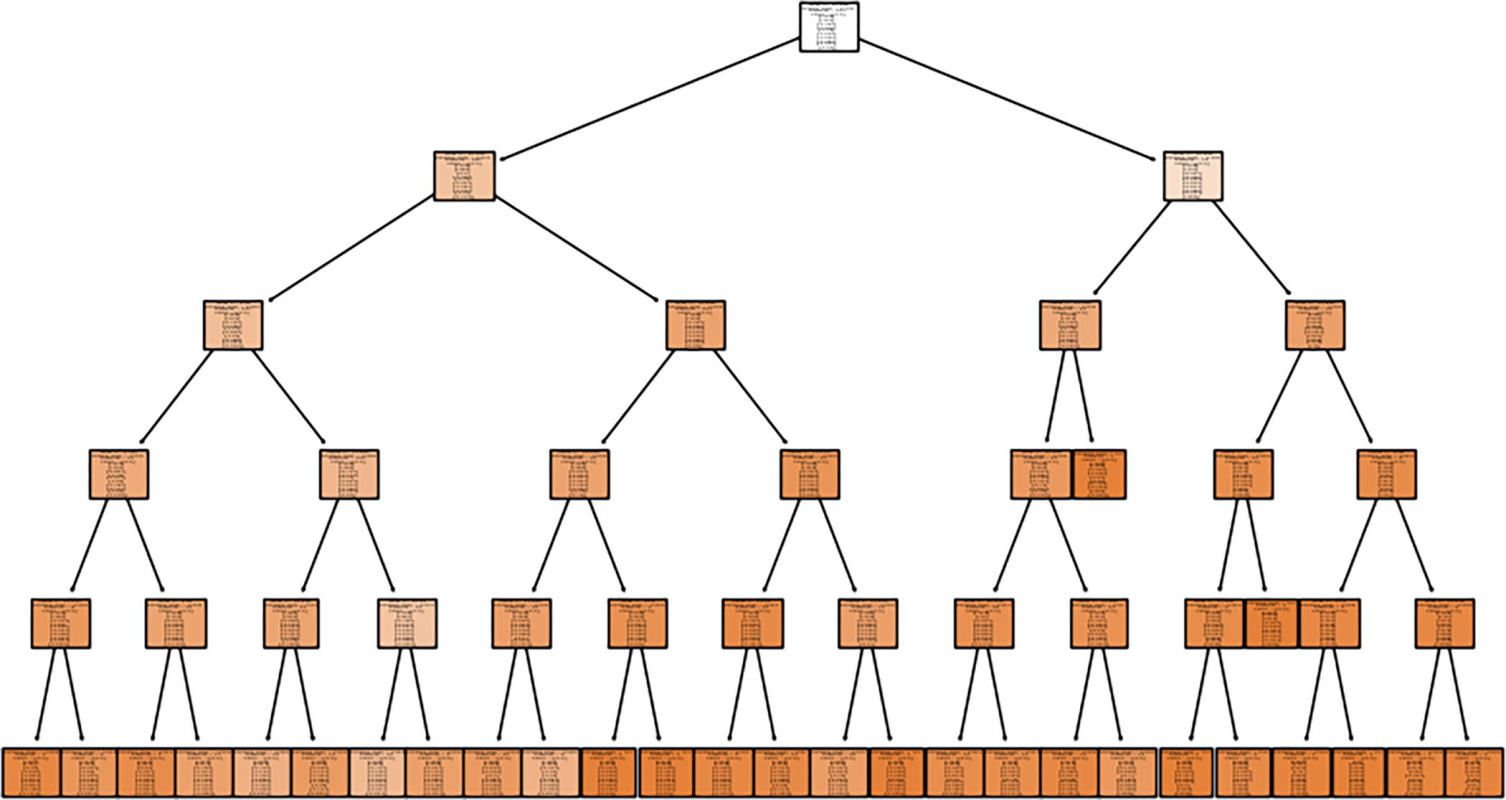
Description of one of the decision trees in the random forest model.

For the RF model, the estimator is set to 50, and the max depth is 5 (Fig 6).

### Performance

Using the least square method, *τ*2 and *τ*1 in the exponential method are 496.72 and 1.5, respectively. The MSE of the RF model is 0.04381, and the MSE of the NN model is 0.04367. However, the MSE of the exponential method is 0.92609, which is much higher than that of the two machine learning models (Fig 6).

**Fig 7:**
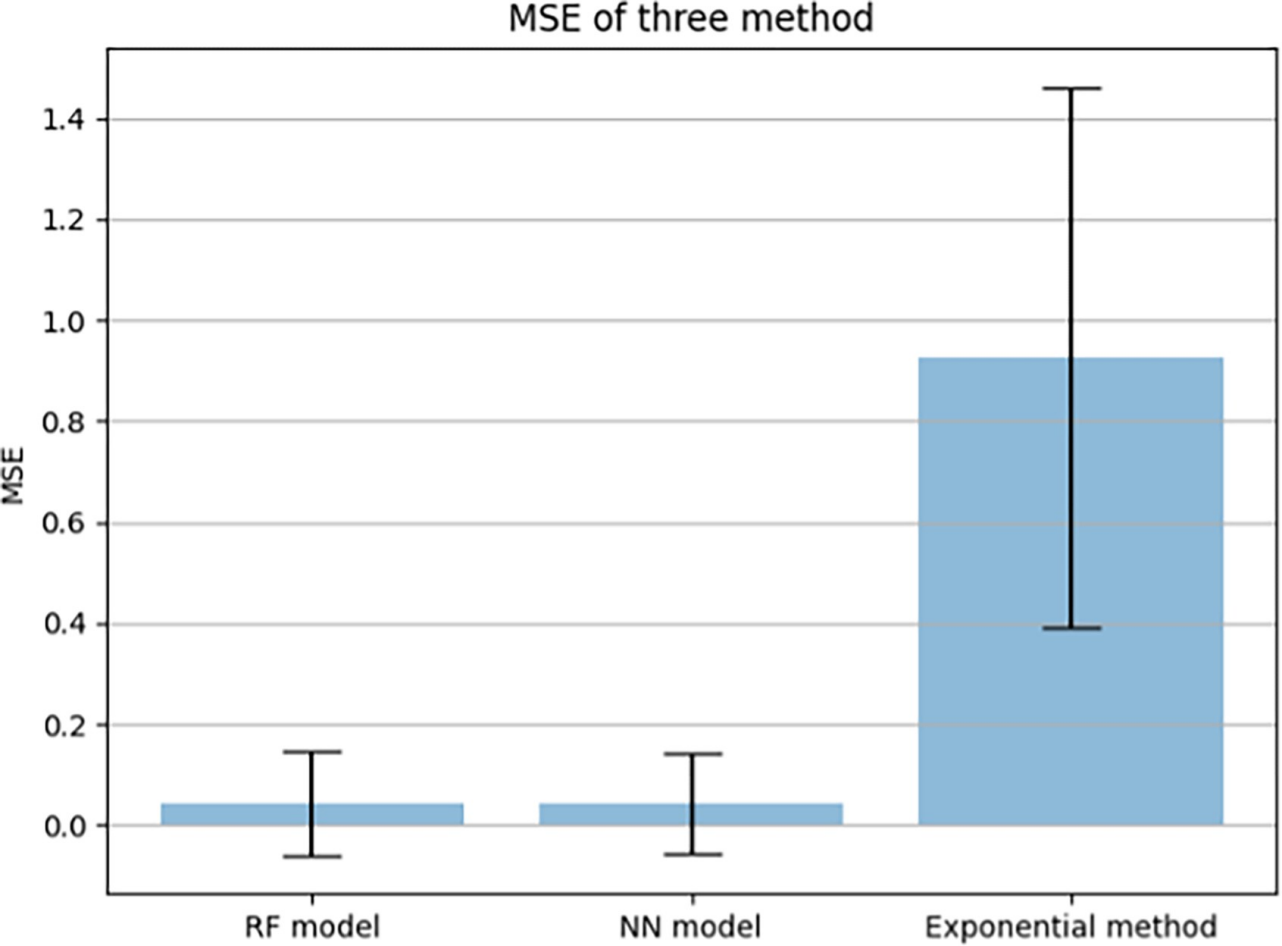
Bar chart of the mean MSE for three methods.

**Fig 8:**
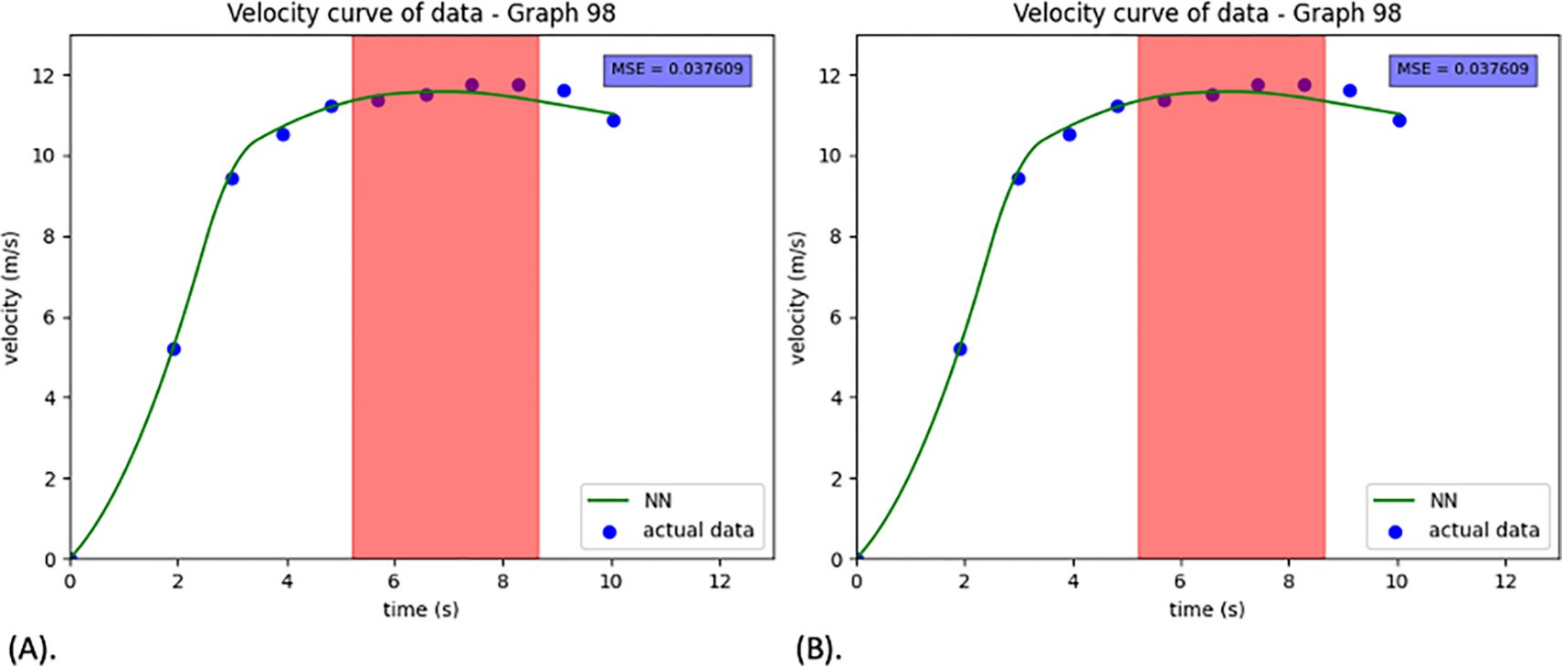
Two samples of NN model curve fitting. Fig 8A is an example of better prediction with a lower MSE. Fig 8B shows an example of poor prediction performance. The red highlighted region represents the maximum phase of the NN modeled curve, including 0.98 * max speed to max speed.

**Fig 9:**
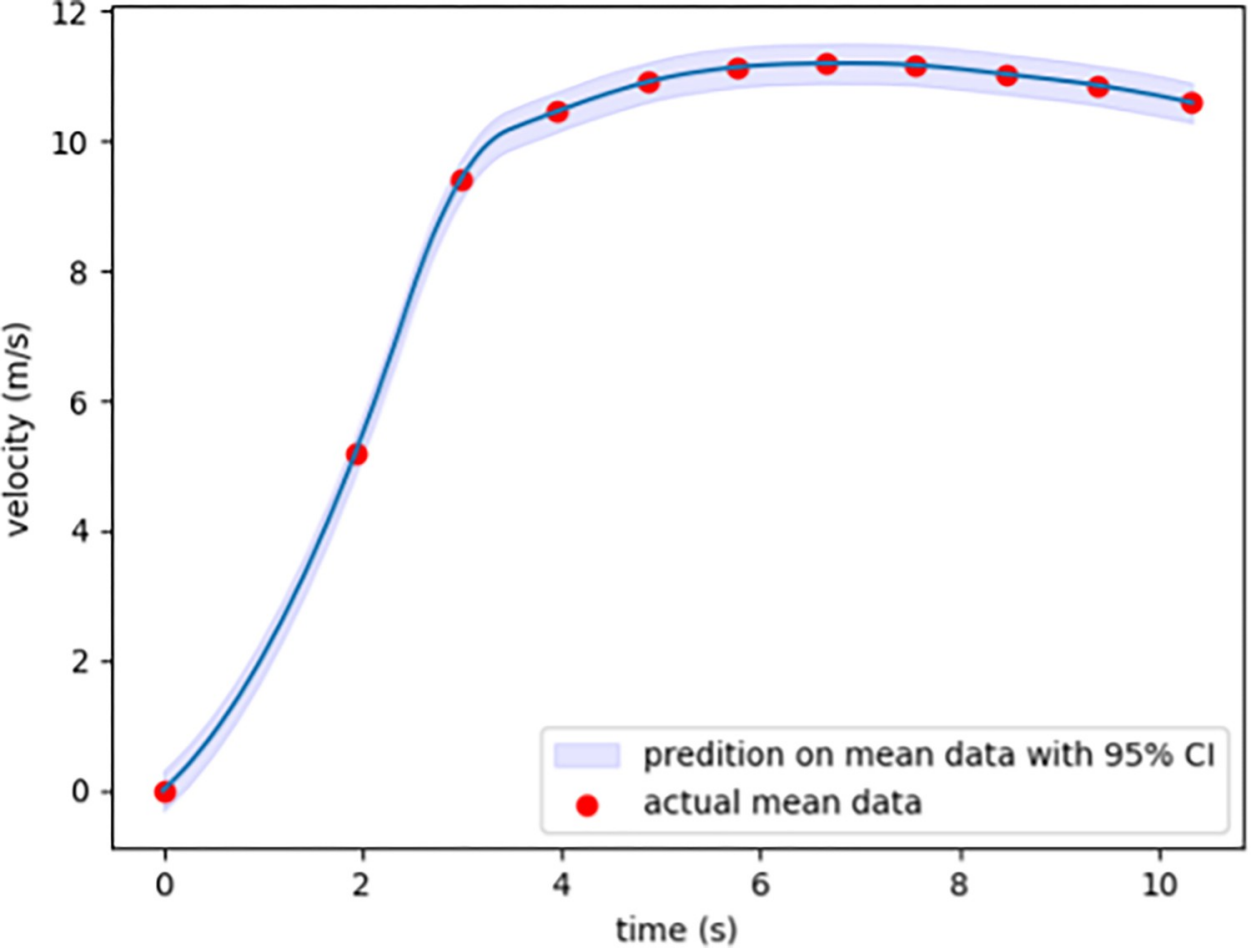
The predicted mean value of each 10m with 95% confidence interval.

The exponential method had a higher MSE and greater standard deviation. Since our NN model had the lowest MSE, the curve fitting is based on the NN model (Fig 7). The predicted mean value of each 10m is shown in Figs 8, 9.

**Fig 10:**
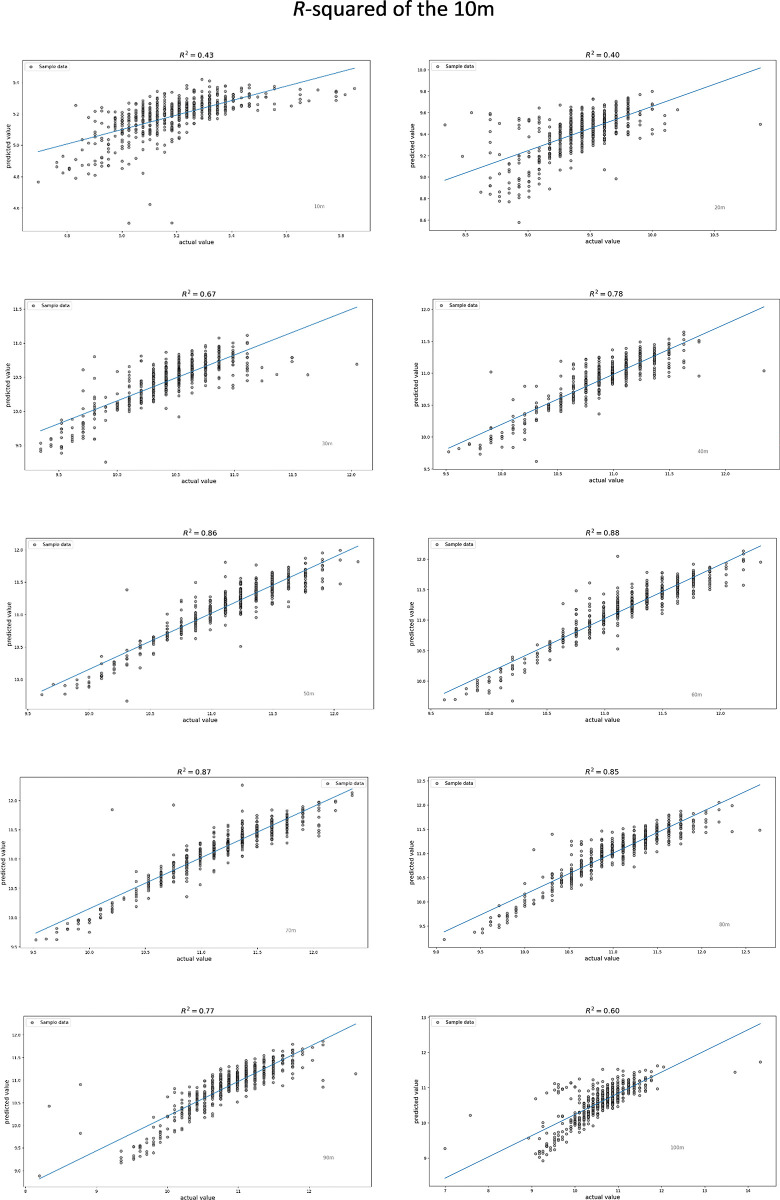
The scatter plot of each 10m with regression line. The x-axis is the actual value and the y-axis is our predicted value.

After selecting the NN model, we calculated the *r*^2^ for each predicted value. Among them, only V_10_ (*r*^2^ = 0.43), V_20_ (*r*^2^ = 0.40), and V_100_ (*r*^2^ = 0.60) exhibited a medium effect size. Conversely, all other targets demonstrated a large effect size. The individual *r*^2^ values are depicted in Fig 10.

### Correlation

**Fig 11:**
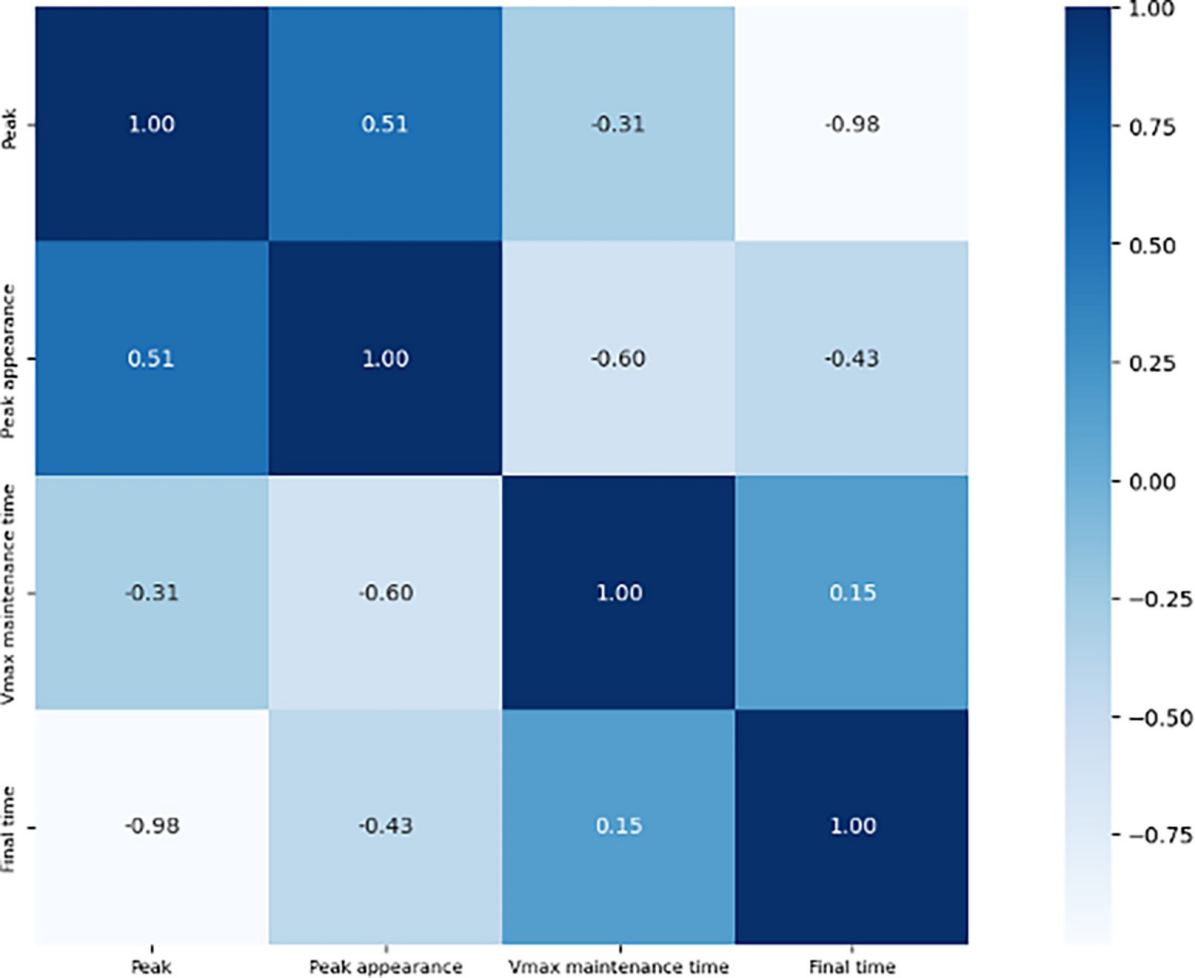
Correlation heatmap of maximum velocity, time of maximum velocity appearance, length of maximum velocity phase, and the final time.

The results of the correlation coefficients for four parameters are shown in Fig 11. Maximum velocity exhibits a nearly perfect negative correlation (*r* = -0.98) with final time. The time of maximum velocity appearance shows a moderate correlation with final time (*r* = -0.43). A very weak correlation relationship is observed between the length of maximum velocity maintenance and final time (*r* = 0.15).

## Discussion

This study provided details on modeling the velocity-time curve in 100m sprinting using machine learning algorithms. The study addresses the limitations of traditional speed models by introducing a method that uses fewer variables to generate more accurate predictions. Data from various international track events from 1987 to 2019 were used, employing Random Forest and Neural Network models. The findings suggest that machine learning can offer new insights into sprint performance, indicating a strong negative correlation between maximum velocity and final time. The findings from our study not only advance the theoretical understanding of sprint performance dynamics but also have direct practical implications. For instance, coaches and athletes can leverage our machine learning models to tailor training programs that specifically target phases of the sprint where the athlete has the most room for improvement, thereby optimizing performance outcomes in sprinting.

The primary findings highlighted ML algorithms’ efficacy in modeling 100m sprint velocity curves. Our findings indicate that ML models, specifically random forest and neural network algorithms, outperform traditional biexponential speed models in predicting the velocity-time (v(t)) curves for sprinters. This is consistent with the work of Horvat and Job (2020) [10], who emphasized the potential of ML in sports outcome prediction, and Ao et al. (2019) [21], who found linear random forest algorithms to be particularly effective for non-linear targets. The performance analysis underscored the superiority of machine learning models in handling non-linear data, as evidenced by the lower mean squared error (MSE) compared to the bi-exponential model [2, 21]. The NN model, in particular, demonstrated the lowest MSE, indicating its potential as a robust tool for sprint performance prediction [10, 23, 24].

The traditional biexponential models, while providing a foundational understanding of sprint dynamics [2, 25], showed significant limitations in our analysis. The traditional equations are primarily regarded as assisting tools for timing mechanics to yield more accurate results [26, 27]. These models required precise data collection and struggled with the initial acceleration and deceleration phases, which are critical in the context of a 100m sprint. The ML models demonstrated a marked improvement in this area, requiring fewer variables and offering a better fit, supporting the hypothesis that ML can streamline the velocity curve modification process [9].

The use of ML also facilitated a more detailed exploration of the relationship between various sprinting parameters, such as the velocity of maximum speed and the timing of its occurrence. The correlations identified in this study between these parameters and the final sprint time have important implications for athlete training and performance analysis. This finding aligns with researchers [5, 6, 28] who have underscored the importance of kinematic analysis in sprint performance optimization. Furthermore, this opens up the possibility of exploring pacing strategies for the 100m sprint. Given the interplay between factors such as the duration of maximum velocity, the magnitude of peak velocity, and their timing, there is potential to delve deeper into optimizing race strategies based on these variables. Coaches can easily identify an athlete’s weaknesses related to the concept of different phases in sprinting [18, 29].

Furthermore, our ML model extends the capacity for understanding an athlete’s performance and condition beyond conventional methods. Historically, studies have employed mathematical analysis to distill parameters from velocity-time (v(t)) curves to assess mechanical aspects and fatigue [2, 30–32]. Our model enhances this approach by enabling the extraction of these parameters more efficiently. This observation can provide valuable insights into the athletes’ conditioning, thereby informing and potentially revolutionizing training practices. Such an implication is not only applied in 100m but also in sports that include sprinting, such as football and rugby [33, 34]. Nonetheless, the application of ML in sports science extends beyond sprint performance prediction. Similar methodologies have been employed to analyze injury prevention strategies [12], athlete selection processes [23], and even game outcome predictions [9]. These studies underscore the versatility and potential of machine learning to revolutionize various aspects of sports science and athlete development.

This study presents a novel approach to predicting 100m sprint performance using ML algorithms, highlighting significant advancements over traditional models. However, it is essential to acknowledge its limitations and outline potential directions for future research. Given the rarity of studies utilizing machine learning to predict sprinting performance and velocity development, it is important to acknowledge several limitations that researchers and coaches must consider when applying these findings in practice. Firstly, our model did not account for various external factors that could influence performance, such as wind speed, types of spike shoes worn, and competition temperature. A comprehensive database incorporating these variables could be established in the future to facilitate the development of more accurate predictive models. These factors are known to significantly impact an athlete’s ability to achieve optimal performance [34, 35]. Specifically, Subsequent research should focus on translating these predictive models into practical tools for coaches and athletes. Developing user-friendly interfaces that allow for the input of individual athlete data and provide tailored training recommendations could bridge the gap between theoretical models and practical applications. Secondly, the data were collected from various reports spanning over 30 years. Given the significant advancements in timing systems over this period, relying solely on recent data may not provide a sufficiently large sample size to yield reliable results. Future studies should aim to include data from a wider demographic of athletes. Junior and master’s level athletes could be included in the study, which could provide insights into performance patterns across different age groups, potentially leading to age-specific training and prediction models. Lastly, future studies should explore the application of ML algorithms in predicting performance in the 200m and 400m sprints. This application would not only validate the models’ applicability across different sprint distances but also contribute to a holistic understanding of sprint performance dynamics.

In summary, the application of ML in this study represents a significant advance in modeling sprint velocity curves. It offers a more accessible and accurate approach for coaches and athletes in the field, providing actionable insights into sprinting performance that were previously obfuscated by the limitations of traditional modeling techniques. This finding could herald a new era of data-driven training methodologies in track and field athletics. In future studies, exploring machine learning models for predicting 100m sprinting performance, specifically in female athletes, could be an intriguing avenue of research, especially when compared with models developed for male athletes. Additionally, investigating predictive models for different sprint distances, such as 200m and 400m, could prove to be a valuable investment.
